# Case Report: Probable CLIPPERS in a patient with cutaneous T-cell lymphoma in remission: 5-year follow-up

**DOI:** 10.3389/fimmu.2026.1892153

**Published:** 2026-08-05

**Authors:** Kun Hong, Lantao Liang, Ruohao Li, Zhijiao Song, Meiling Zhang, Hui Bu, Yu Zhang

**Affiliations:** 1 Department of Neurology, The Second Hospital of Hebei Medical University, Shijiazhuang, Hebei, China; 2 Neurological Laboratory of Hebei Province, Shijiazhuang, Hebei, China

**Keywords:** autoimmune, CLIPPERS, MOG-IgG, responsive to steroids, T-cell lymphoma

## Abstract

Chronic lymphocytic inflammation with pontine perivascular enhancement responsive to steroids (CLIPPERS) may be associated with lymphoma, although the underlying relationship between these entities remains unclear. While the diagnosis is centered on specific imaging characteristics and can be difficult to establish, CLIPPERS typically responds robustly to corticosteroid therapy. We report a 60-year-old woman who presented with memory and cognitive impairment 4 years after achieving complete remission from cutaneous T-cell lymphoma. Brain MRI revealed atypical CLIPPERS features, including scattered enhancing lesions in both supratentorial and infratentorial regions. During a subsequent relapse, transient low-titer serum anti-myelin oligodendrocyte glycoprotein antibodies (titer 1:32) were detected, which later reverted to negative. Initial PET/CT demonstrated transient hypermetabolic splenic lesions suspicious for lymphoma involvement, which resolved on follow-up imaging. Subsequently, new skin lesions with T-cell atypia developed; however, skin biopsy findings, CSF T-cell receptor gene rearrangement studies, and clinical evaluations did not provide evidence supporting lymphoma recurrence or CNS involvement. After comprehensive evaluation and exclusion of alternative diagnoses, follow-up brain MRI revealed characteristic “pepper-like” pontocerebellar enhancing lesions. In combination with a favorable response to high-dose corticosteroids, these findings supported a diagnosis of probable CLIPPERS. This case highlights the complex interplay among CLIPPERS, potential neoplastic states, and transient autoantibodies during long-term follow-up, underscoring the need for prolonged clinical surveillance for possible lymphoma recurrence. It also underscores the diagnostic complexity of CLIPPERS in patients with prior lymphoma and suggests that transient low-titer MOG-IgG seropositivity should be interpreted cautiously in the absence of CSF positivity or a typical MOGAD phenotype.

## 1 Introduction

Chronic lymphocytic inflammation with pontine perivascular enhancement responsive to steroids (CLIPPERS) was initially reported by Pittock et al. in 2010, who summarized eight patients presenting with subacute gait ataxia and diplopia; neuroimaging revealed characteristic areas of punctate and curvilinear gadolinium enhancement with a “pepper-like” appearance, predominantly in the pons and cerebellum. Brain biopsy was performed in four, which showed perivascular lymphocytic infiltration within the white matter. All patients exhibited symptomatic improvement after corticosteroid treatment. Post-treatment neuroimaging demonstrated a reduction in the number as well as in the size of lesions. However, long-term maintenance immunosuppressive therapy was required. Six patients experienced relapse when steroid dose reduction or discontinuation was attempted (1). While the pathogenesis of CLIPPERS remains incompletely understood, it is classified as an autoimmune-mediated inflammatory disorder. Its characteristic of perivascular inflammation has led to the organ-specific autoantigen hypothesis. Other proposed disease mechanisms include virus-induced autoimmunity and a Th17 cell-mediated pathway (1, 2).

The etiology of CLIPPERS has not been elucidated. However, it frequently occurs after viral infections (chronic hepatitis B, Epstein-Barr, SARS-CoV-2, varicella-zoster) and influenza vaccination (3–6). Ortega et al. described a patient with multiple sclerosis who developed CLIPPERS following the withdrawal of natalizumab (7). In a study of 140 patients with CLIPPERS, the prevalence of malignancy was 16% (8). The most common associated malignancy is central nervous system (CNS) lymphoma, followed by renal cell carcinoma, thyroid cancer, malignant nerve sheath tumor, breast cancer, prostate cancer, and adrenal adenoma. In a retrospective analysis of 88 CLIPPERS patients, 10 had lymphoma (8–10).

There appears to be no clear temporal relationship between the onset of CLIPPERS and lymphoma—in one case, CLIPPERS appeared 11 years after a cured Hodgkin lymphoma; in another, Hodgkin lymphoma was diagnosed 2 years after the onset of CLIPPERS (5, 11). Additionally, T-cell lymphoma has been documented 5 to 10 years after CLIPPERS onset, and CNS B-cell lymphoma has been observed several years after a CLIPPERS diagnosis. Nonetheless, CLIPPERS is clearly closely associated with lymphoma (12–15). The question is whether it is a truly distinct new disease entity, or rather a syndrome encompassing different, potentially overlapping conditions and their prodromal stages. We report a case of CLIPPERS occurring 4 years after complete remission of cutaneous T-cell lymphoma (CTCL). The patient has been followed for 5 years since the onset of CLIPPERS.

## 2 Case presentation

A 60-year-old woman had been diagnosed with CTCL in 2008 and achieved complete remission in 2016 after approximately 8 years of treatment with interferon, immunosuppressants, and ultraviolet phototherapy. Four years later, in 2020, she presented with fatigue, memory impairment, hypersomnia, reduced verbal output, and vomiting. Her memory impairment was so severe that she had forgotten to turn off the stove after boiling water and was unable to account for other recent activities. Positron emission tomography (PET)/computed tomography (CT) showed an enlarged spleen with slightly heterogeneous density focal nodular hypermetabolism, suggesting a high probability of lymphoma (Deauville score, 4); however, splenic biopsy was not performed, precluding histopathological confirmation. Neurological examination revealed severe impairments in calculation, memory, executive function, and orientation. Her cranial nerves were intact. Sensation and coordination were normal. She exhibited generalized weakness in all four limbs. Deep tendon reflexes were hyperactive throughout. Her modified Rankin scale score was 3. Rapidly progressive dementia was diagnosed.

**Figure 1:**
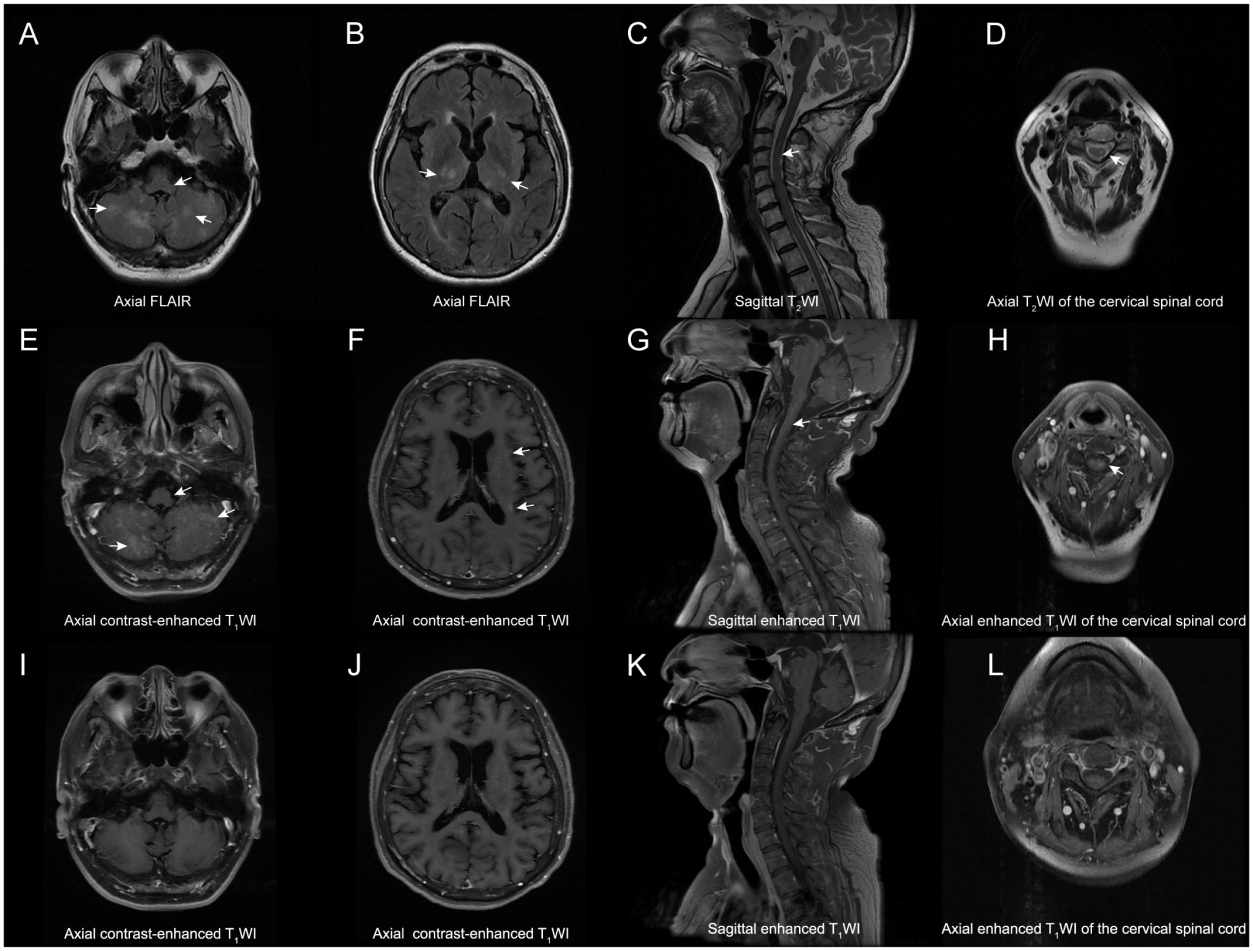
Brain and spinal cord MRI at disease onset **(A–H)** and post-treatment **(I–L)**. **(A, B, E, F)** Multiple slightly hyperintense FLAIR signals with enhancement are present in the brainstem, cerebellar gray and white matter **(A, E)**, the bilateral subcortical and deep cerebral white matter, thalami **(B, F)**. **(C, D, G, H)** Multiple small patchy slightly hyperintense T_2_WI signals with enhancement in the cervical spinal cord parenchyma. **(I, J)** Reduction in the number and size of lesions in the bilateral cerebral hemispheres, brainstem, and cerebellar hemispheres. **(K, L)** No abnormal signal or abnormally enhancing lesions are seen in the cervical spinal cord. MRI, magnetic resonance imaging; T_1_WI, T_1_-weighted imaging; T_2_WI, T_2_-weighted imaging; FLAIR, fluid-attenuated inversion recovery.

A broad serologic workup was negative for human immunodeficiency virus, rapid plasma reagin, antinuclear antibodies, and abnormalities in kidney and liver markers; levels of vitamin B12, folate, electrolytes, and thyroid-stimulating hormone were normal. Routine cerebrospinal fluid (CSF) analyses were also within normal limits. CSF protein was elevated (50 mg/dL). Serum and CSF aquaporin-4 (AQP4) and myelin oligodendrocyte glycoprotein (MOG) immunoglobulin G (IgG) antibodies were undetectable, as were serum and CSF autoimmune/paraneoplastic encephalitis panels. No oligoclonal bands were detected. Electroencephalography showed background slowing with diffuse polymorphic delta and theta waves. A low burden of spike-and-wave and sharp-wave complexes was seen maximally over the bilateral temporal regions, including the sphenoidal electrodes. Brain MRI showed multiple punctate and patchy T_2_-weighted (T_2_WI) and fluid-attenuated inversion recovery (FLAIR) hyperintensities involving both supratentorial and infratentorial regions, including the subcortical and deep white matter, brainstem, thalami, cerebellar hemispheres, and medulla oblongata, with prominent post-contrast enhancement (**Figures 1A, B, E, F**). On diffusion-weighted imaging (DWI), some lesions in the bilateral cerebral hemispheres were mildly hyperintense, with corresponding isointensity on apparent diffusion coefficient (ADC) maps. Brain magnetic resonance angiography (MRA) revealed no vascular abnormalities. Spinal MRI showed multiple small patchy T_2_-hyperintense lesions in the cervical spinal cord, with multiple punctate and linear areas of enhancement and no mass effect (**Figures 1C, D, G, H**). MR spectroscopy (MRS) of the right periventricular and insular regions was performed with an echo time (TE) of 144 ms, with the spectroscopy voxel centrally positioned within the enhancing component of the lesion. It demonstrated an elevated choline peak, a stable creatine peak, and a reduced N-acetylaspartate peak, yielding a choline/creatine (Cho/Cr) ratio of 0.68–1.45 and a choline/N-acetylaspartate (Cho/NAA) ratio of 1.03–1.18. No lipid or lactate peaks were observed. The patient received a course of high-dose intravenous methylprednisolone (500 mg/day for 5 days), which was tapered to oral prednisone (1 mg/kg/day). Her symptoms gradually improved, with notable recovery in recent memory and calculation abilities. Follow-up MRI of the brain showed a significant reduction in both the size and the enhancement of the previously noted lesions (**Figures 1I, J**). Upon follow-up at 3 months, repeat imaging showed complete resolution of the cervical cord lesions (**Figures 1K, L**); clinically, she had also improved (modified Rankin scale score 1).

**Figure 2:**
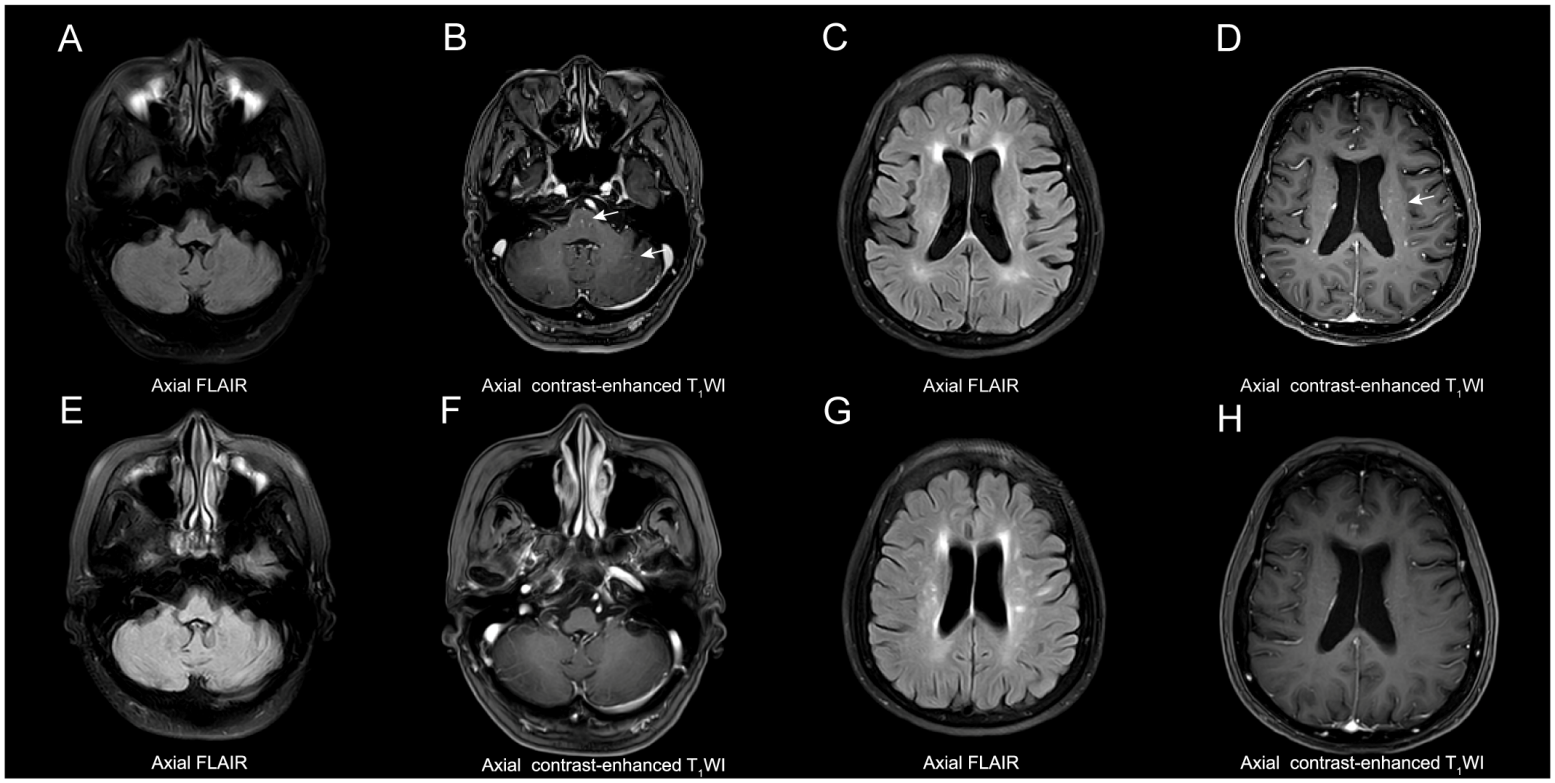
Brain MRI images at first relapse **(A–D)** and during remission **(E–H)**. **(A–D)** Multiple areas of mild FLAIR hyperintensity in the bilateral cerebral hemispheres, brainstem, and cerebellum, with enhancement. **(E–H)** Reduced extent or disappearance of multiple enhancing lesions in the cerebral hemispheres, brainstem, and cerebellum. MRI, magnetic resonance imaging; T_1_WI, T_1_-weighted imaging; FLAIR, fluid-attenuated inversion recovery.

Four years later, she presented with transient loss of consciousness, worsening recent memory loss, and generalized limb weakness. She denied seizures. Brain MRI revealed multiple enhancing lesions in both cerebral hemispheres and the brainstem and cerebellum, with DWI demonstrating punctate hyperintensities in the pons and left temporal lobe, corresponding to isointensity on ADC maps (**Figures 2A–D**). CSF analysis was notable for an elevated protein level (87 mg/dL). Serum MOG-IgG was positive (1:32) on two separate tests by live cell-based assay (live CBA), performed at V-Medical Laboratory Co., Ltd., whereas CSF testing was consistently negative. Testing for paraneoplastic, glial fibrillary acidic protein (GFAP), and AQP4 antibody panels was negative in both the serum and CSF. Intravenous methylprednisolone (500 mg daily for 3 days) followed by rituximab (400 mg; infusion discontinued because of infusion-related chills and fever) was initiated for recurrent neurological symptoms, which resulted in symptom resolution. Two months later, follow-up MRI demonstrated significant reduction or complete resolution of the brain lesions (**Figures 2E–H**).

**Figure 3:**
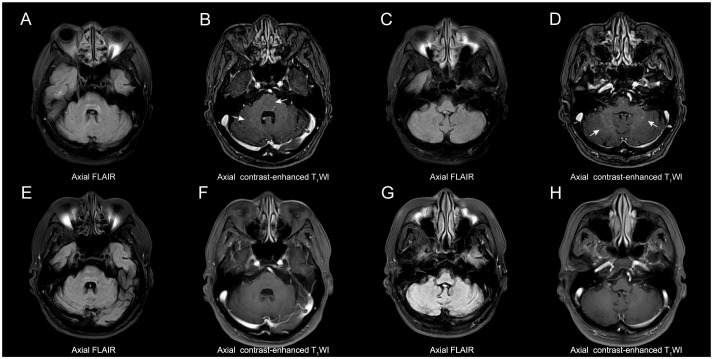
Brain MRI images at second relapse **(A–D)** and during remission **(E–H)**. **(A–D)** Increased number of scattered punctate and linear enhancing foci in the pons and cerebellar hemispheres. **(E–H)** Decreased number of scattered small punctate and linear enhancing foci in the brainstem and cerebellar hemispheres. MRI, magnetic resonance imaging; T_1_WI, T_1_-weighted imaging; FLAIR, fluid-attenuated inversion recovery.

**Figure 4:**
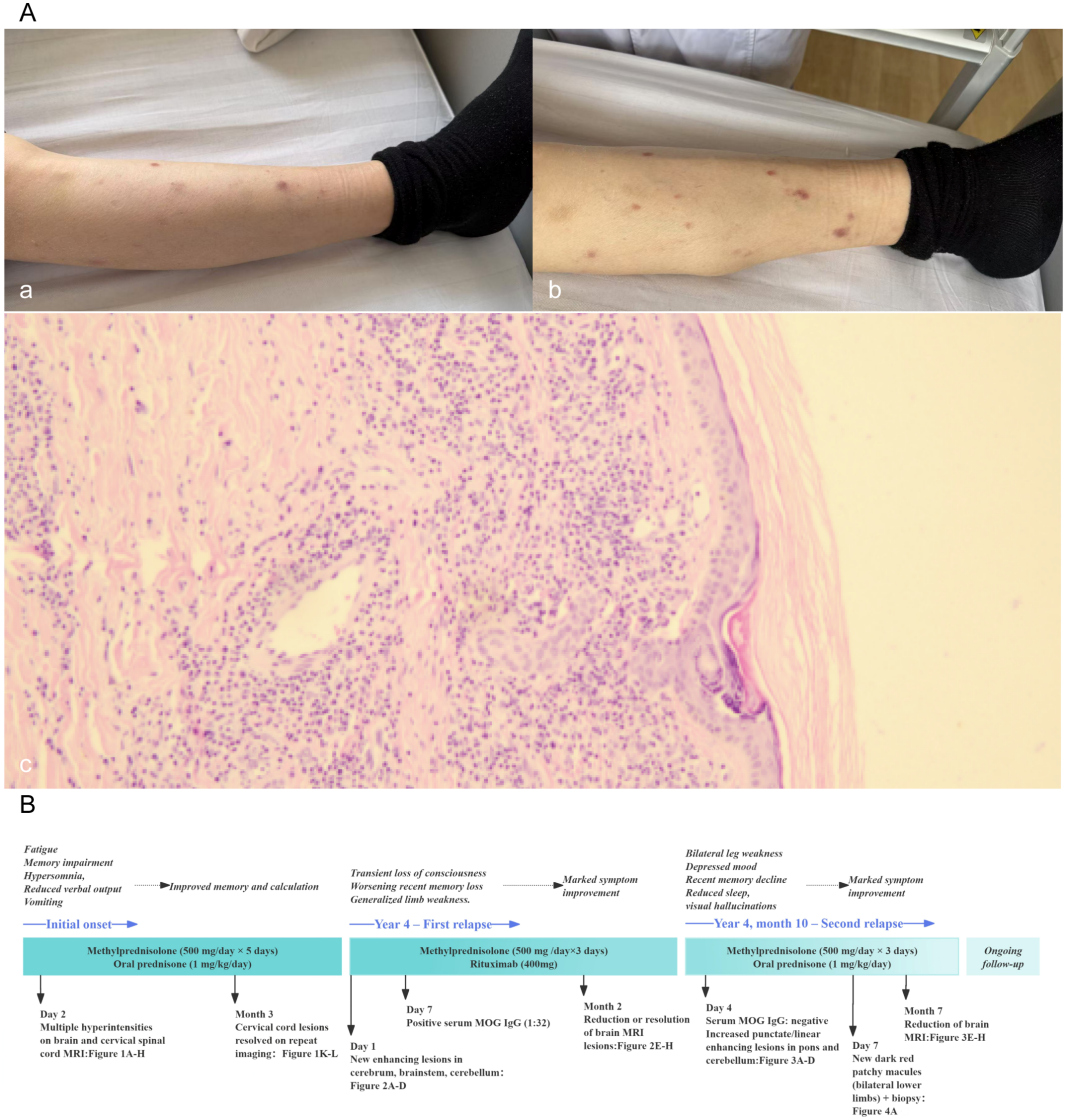
**(A)** Gross image and histopathology of the patient’s skin: (a, b) Scattered dark red macules on both lower limbs and two indurated erythematous plaques on the left lower leg. (c) Subcutaneous irregular nested proliferation of atypical T-lymphocytes (left lower leg biopsy). **(B)** The timeline of relevant results and interventions during the diagnosis and treatment of the patient.

Ten months later, the patient developed bilateral lower extremity weakness and an unsteady gait, accompanied by depressed mood, anhedonia, and irritability. She was then hospitalized, during which, her family members reported significant recent memory decline, agitation, reduced nighttime sleep, and visual hallucinations. MRI revealed an increase in scattered punctate and linear enhancing lesions within the pons and bilateral cerebellar hemispheres, with no abnormal signal detected in the cervical spinal cord. DWI demonstrated no abnormal hyperintense signal in the brain parenchyma. (**Figures 3A–D**). These findings were consistent with CLIPPERS. CSF analysis showed 18 nucleated cells/µL and an elevated protein level (84 mg/dL). Serum MOG IgG was negative. Her physical examination revealed new skin lesions consisting of scattered, dark red, patchy macules on both lower limbs and two slightly infiltrated erythematous plaques on the left lower leg (**Figures 4A**, a, b). A biopsy from the left lower leg demonstrated atypical T-lymphoid hyperplasia with a subcutaneous, irregular nested proliferation of T-lymphocytes. The immunophenotype was CD3(+), CD5(+), CD7(+), and CD43(+), with partial expression of CD4 and CD8; GrB was scattered positive, and Ki-67 was approximately 20%. Cells were negative for ALK, CD2, CD20, CD30, CD56, and EBER (**Figures 4A**, c). T-cell receptor (TCR) gene rearrangement analyses of both skin biopsy and cerebrospinal fluid samples were negative. Regrettably, the original raw data from the skin TCR gene rearrangement assay were not available, as the test was performed at an external institution. A repeat whole-body PET/CT, performed approximately 4.75 years after the initial examination, demonstrated complete resolution of the previously noted splenic hypermetabolic nodules (**Supplementary Table 1**) and identified only a small focus of mildly increased metabolic activity in the posterior aspect of the right lower leg.

After a 3-day pulse of intravenous methylprednisolone (500 mg/day), the patient’s symptoms improved. To prevent relapse, oral prednisone was initiated on discharge at 1 mg/kg/day, and then tapered. At the most recent follow-up, brain MRI demonstrated reduction of multiple scattered small dot-like and linear enhancing lesions in the brainstem and bilateral cerebellar hemispheres relative to prior imaging, and the patient was independent in basic routine activities (modified Rankin scale score 1), with recovered memory and no further relapses (**Figures 3E–H**). The relevant diagnosis and treatment results of the patient are shown in **Figure 4B**.

## 3 Discussion and conclusions

This report describes a complex case of probable CLIPPERS that developed following complete remission of CTCL, with a longitudinal follow-up of five years. Several features made the diagnostic evaluation particularly challenging, including cognitive impairment as the initial presenting manifestation, extensive supratentorial and infratentorial lesions on MRI, transient low-titer serum MOG-IgG positivity during the disease course, and the subsequent development of cutaneous lesions with atypical T-cell hyperplasia. The coexistence of these findings posed diagnostic challenges regarding CLIPPERS, occult lymphoma, and the clinical significance of dynamic autoantibody positivity. This case highlights the importance of comprehensive differential diagnosis and long-term multidisciplinary surveillance in patients with atypical manifestations of CLIPPERS.

According to the revised diagnostic criteria proposed by Tobin et al. (2017), this patient met the criteria for probable CLIPPERS (9), as detailed in **Supplementary Table 2**. Notably, the diagnostic criteria were not fulfilled until the first relapse, when the characteristic clinical manifestations and pontocerebellar MRI features became evident. Therefore, the diagnosis was established retrospectively rather than at the index presentation. The diagnosis was supported by characteristic clinical and radiological features, including brainstem, cerebellar, and cognitive involvement with a relapsing–remitting course, punctate and curvilinear pontocerebellar enhancement with a typical “pepper-like” appearance, a robust response to corticosteroids, and evaluation of major mimics including CNS lymphoma. Brain biopsy was considered at the initial presentation but was ultimately not performed because the patient declined the procedure. Therefore, the diagnosis remained classified as probable CLIPPERS, and the absence of histopathological confirmation remains an important limitation of this case.

This case exhibited several atypical clinical and radiological features that merit further discussion. Firstly, the patient presented predominantly with cognitive impairment, including memory, calculation, and executive dysfunction, rather than the classic brainstem and cerebellar manifestations of CLIPPERS, such as dysarthria, ataxia, and diplopia. Previous studies have suggested that cognitive dysfunction is an easily overlooked manifestation of CLIPPERS, particularly in patients with prominent supratentorial involvement (16, 17). Secondly, the patient’s initial brain MRI demonstrated extensive infratentorial and supratentorial involvement, including the bilateral subcortical and deep cerebral white matter as well as the thalami, rather than the typical pontocerebellar distribution. This “supratentorial-predominant” presentation is sometimes termed “supratentorial lymphocytic inflammation with parenchymal perivascular enhancement responsive to steroids (SLIPPERS)” (18–20). Notably, characteristic pontocerebellar punctate enhancement emerged only during subsequent relapses in our patient, suggesting that the radiological phenotype may evolve over time, and early atypical imaging should not exclude CLIPPERS. Thirdly, MRI revealed multiple small enhancing lesions in the cervical cord. While spinal cord involvement is uncommon in CLIPPERS, prior reports (21, 22) present diagnostic challenges regarding MOGAD, multiple sclerosis, and lymphoma. Importantly, lesion resolution following corticosteroid therapy supports the reversible, inflammatory process to some extent, although this finding does not completely exclude an underlying neoplastic process.

Distinguishing CLIPPERS from lymphoma represents the primary diagnostic challenge in this case, particularly in the absence of brain biopsy. Primary or systemic lymphoma can mimic CLIPPERS, often responding transiently to steroids (12, 23) and showing potential evolution over time (24). Although the patient presented with risk factors predictive of underlying lymphoma, including a history of CTCL, splenic hypermetabolism, hyperreflexia, elevated CSF protein, and relapse (25), 5-year follow-up did not reveal clinical or radiological evidence suggestive of progressive malignancy. Two PET/CT scans showed splenic lesion resolution without new foci. However, the interpretation of these findings as evidence against lymphoma is limited because prior corticosteroid and rituximab treatment may have confounded the PET/CT results. Comprehensive analyses (CSF TCR gene rearrangement, repeated skin biopsies) did not demonstrate clonal lymphoproliferation. Furthermore, neuroimaging lacked malignant features (ring enhancement, mass effect), and no disease progression occurred.

Alternative explanations for the co-occurrence of CTCL and CLIPPERS include a paraneoplastic process or pure coincidence. Nakamura et al. have proposed aberrant proliferation of CD4+ T cells as a potential mechanism linking lymphoma with CLIPPERS (26). This association, although rarely reported, has been increasingly recognized in recent studies (8, 27). CTCL and CLIPPERS may share a common immunopathological basis involving T-cell-mediated inflammatory responses. CTCL is a T-cell-derived malignancy (28), whereas the pathological hallmark of CLIPPERS is dense perivascular T-lymphocytic infiltration (1). In our patient, skin biopsy demonstrated atypical T-cell proliferation expressing CD3, CD5, CD7, and CD43, with partial co-expression of CD4 and CD8, which may represent a manifestation of persistent T-cell immune dysregulation. Accordingly, CLIPPERS in this case may reflect immune dysregulation in the setting of prior CTCL rather than a direct lymphomatous process. We acknowledge, however, that CD2 loss and a Ki-67 index of approximately 20% raised concern for a possible neoplastic process (29), and in view of the imperfect sensitivity of TCR-PCR clonality (30), recurrent CTCL cannot be completely excluded. Therefore, long-term clinical and radiological surveillance is essential. Future studies incorporating histopathological confirmation and longitudinal immune profiling are warranted to further elucidate the relationship between CLIPPERS and lymphoproliferative disorders. Nonetheless, in the absence of brain biopsy and definitive evidence of active lymphoma, these interpretations remain speculative.

The diagnosis of CLIPPERS is fundamentally one of exclusion. Given the distinctive clinical and radiological features observed in this patient, several important alternative diagnoses required careful consideration, and the corresponding diagnostic findings are summarized in **Supplementary Table 3**.

Primary central nervous system lymphoma (PCNSL) represented the most important differential diagnosis in this case. Restricted diffusion with corresponding low ADC values is a well-recognized imaging hallmark of PCNSL and an important feature distinguishing it from CLIPPERS. Marked diffusion restriction reflects the high cellularity of lymphoma and has been reported in approximately 90% of PCNSL cases (24, 31, 32). Furthermore, ADC values are inversely correlated with tumor cellularity and proliferative activity, making diffusion imaging a valuable biomarker for the diagnosis of PCNSL and the detection of disease relapse (33). In addition, typical imaging features of PCNSL include ring-like enhancement, mass effect, and elevated lipid/lactate peaks on MRS (31, 34, 35). In contrast, our patient demonstrated only mildly increased signal intensity on DWI with corresponding isointense ADC maps, without ring enhancement, mass effect, or abnormal lipid/lactate peaks on MRS (36, 37). Moreover, two PET/CT scans revealed no evidence of metabolically active lymphoma, and CSF TCR gene rearrangement analysis was negative. Nevertheless, the absence of perfusion imaging (DSC/DCE) in this case represents a limitation of the diagnostic evaluation.

Another important diagnostic consideration was secondary CNS involvement by CTCL, given the patient’s history. Although uncommon, CNS dissemination of CTCL has been well documented and may occur several years after the initial diagnosis (38), even in the absence of overt systemic relapse (39–41). Reported manifestations include rapid and sometimes fluctuating cognitive decline, seizures, headache, cranial nerve palsy, and transient responsiveness to corticosteroids. Brain MRI may be unremarkable or demonstrate T2 hyperintense lesions involving the pons and right middle cerebellar peduncle. These reported clinical and imaging features show some overlap with those observed in our patient.

Neither CSF nor skin TCR gene rearrangement analyses demonstrated evidence of clonal T-cell proliferation, and biopsy of the newly developed skin lesions showed atypical T-cell proliferation without definitive evidence of CTCL recurrence. During 5 years of follow-up, two PET/CT scans showed no evidence of systemic lymphoma recurrence, and the patient remained clinically stable without evidence of lymphoma progression. Such a clinical course is less typical of CNS involvement by CTCL, which is generally associated with a poor prognosis (41, 42). Serial MRI evolved toward the characteristic pepper-like pontocerebellar enhancement of CLIPPERS rather than a progressive mass-like or infiltrative pattern. Nevertheless, in the absence of a brain biopsy, secondary CNS involvement by CTCL could not be definitively excluded. Overall, the available findings were considered more consistent with probable CLIPPERS than with secondary CNS involvement by CTCL.

Antibody-negative autoimmune encephalitis (AE) was also considered in this patient, who presented with cognitive impairment, neuropsychiatric symptoms (irritability and visual hallucinations), and bilateral temporal epileptiform discharges on EEG along with negative serum and CSF neuronal autoantibody panels. Brain MRI demonstrated diffuse involvement of the brainstem, cerebellum, cerebral white matter, and cervical spinal cord rather than the characteristic mesial temporal lobe and hippocampal abnormalities of limbic encephalitis. The evolution of MRI findings toward characteristic punctate pontocerebellar enhancement during subsequent relapses, together with negative CSF oligoclonal bands, favored CLIPPERS over antibody-negative AE (43).

Despite increasing reports of overlap between CLIPPERS and MOG antibody-associated disease (MOGAD) (44–46), low-titer serum MOG-IgG positivity in an atypical clinical phenotype should be interpreted with caution because its positive predictive value is limited and false-positive results may occur (47). A summary of the literature on reported CLIPPERS cases with MOG-IgG positivity, lymphoma association, and other mimics is provided in **Supplementary Table 4**. In our patient, negative CSF MOG-IgG and the absence of hallmark MOGAD features, such as optic neuritis or longitudinally extensive transverse myelitis, suggest that the transient serum MOG-IgG positivity was more likely an inflammatory bystander phenomenon rather than evidence of MOGAD (48, 49).

Autoimmune glial fibrillary acidic protein (GFAP) astrocytopathy was considered because of the combined brain and spinal cord involvement; however, both serum and CSF GFAP antibodies were negative, and brain MRI lacked the characteristic radial perivascular enhancement pattern (50, 51). Primary CNS vasculitis (PCNSV) was considered unlikely given the absence of stroke-like episodes, acute infarction on DWI/ADC, and vascular abnormalities on MRA (24, 52). Neurosarcoidosis was considered unlikely because chest CT was unremarkable and there was no evidence of hilar lymphadenopathy or uveitis (53). Intravascular lymphoma was considered because of the patient’s cognitive impairment. However, the absence of infarct-like lesions on diffusion imaging, clonal lymphoproliferation in skin biopsy specimens or CSF, and systemic manifestations during 5 years of follow-up argued strongly against this diagnosis (54, 55). Collectively, these findings further supported the diagnosis of probable CLIPPERS after systematic exclusion of its major inflammatory and neoplastic mimics.

The recommended initial treatment for CLIPPERS is high-dose corticosteroids, typically intravenous methylprednisolone 1 g daily for 5 days, followed by a slow taper of oral prednisone starting at 1 mg/kg per day. Studies have suggested that the mean annualized relapse rate in CLIPPERS patients is 0.5. Among patients on long-term oral corticosteroids, no relapses or disease progression have been observed in those taking at least prednisone 20 mg daily (56). Long-term corticosteroid therapy is essential; however, the optimal dose and duration of treatment remain unclear. Considering the side effects of steroids, other immunosuppressive agents such as methotrexate, mycophenolate mofetil, or azathioprine can be introduced once the enhancing lesions have completely resolved. Second-line immunomodulatory agents have also been proposed as alternatives for maintenance therapy. Anti-CD20 monoclonal antibodies (such as rituximab and ocrelizumab), interleukin-6 receptor antagonists (tocilizumab), and interferon β-1a have all demonstrated efficacy (57–59). Leflunomide and cladribine can also enable sustained clinical and radiological remission in CLIPPERS, serving as long-term replacements for steroids (60, 61). Our patient was initially treated with corticosteroids and experienced her first relapse approximately 3 years after discontinuation. The relapse was managed with corticosteroids combined with rituximab. However, a second relapse occurred as the corticosteroids were tapered and discontinued. Following the second relapse, a long-term maintenance dose of 5 mg prednisone was instituted, and no further relapses have occurred. Our findings suggest that, when CD20-depleting therapy alone is insufficient, maintenance treatment with low-dose corticosteroids may be considered as a potential strategy for relapse prevention in patients with CLIPPERS. However, this observation is based on a single case and should be interpreted with caution. Larger prospective studies are needed to determine the efficacy, optimal duration, and patient selection criteria for maintenance corticosteroid therapy in CLIPPERS.

In conclusion, this case illustrates the diagnostic complexity of probable CLIPPERS in the setting of previous cutaneous T-cell lymphoma and highlights the value of systematic differential diagnosis and long-term follow-up. The absence of brain biopsy, advanced perfusion MRI, quantitative ADC measurements, and longitudinal cerebrospinal fluid assessments, along with the potential confounding effects of treatment, limits diagnostic certainty. Further studies incorporating histopathological confirmation and standardized longitudinal immune evaluation are needed to better define atypical CLIPPERS.

## Data Availability

The original contributions presented in the study are included in the article/**Supplementary Material**. Further inquiries can be directed to the corresponding authors.
