# Supplementary material for: Case Report: Probable CLIPPERS in a patient with cutaneous T-cell lymphoma in remission: 5-year follow-up
Source: Front Immunol. 2026 Aug 5;17:1892153. doi: 10.3389/fimmu.2026.1892153 (PMC13485524; doi:10.3389/fimmu.2026.1892153)
Supplement: Supplementary file 1 [file Table1.docx]

Case Report: Probable CLIPPERS in a patient with cutaneous T-cell lymphoma in remission: 5-year follow-up

Kun Hong^1,2†^ Lantao Liang^1,2†^ Ruohao Li^1,2^ Zhijiao Song^1,2^ Meiling Zhang^1,2^ Hui Bu^1,2^* Yu Zhang^1,2^*

^1^Department of Neurology, The Second Hospital of Hebei Medical University, Shijiazhuang, Hebei,050000, China

^2^Neurological Laboratory of Hebei Province, Shijiazhuang , Hebei,050000, China

***Correspondence:**
†These authors contributed equally: Kun Hong, Lantao Liang

*Correspondence and requests for materials should be addressed to: 26500825@hebmu.edu.cn (H Bu), 28502360@hebmu.edu.cn (Y Zhang)

| **Supplementary Table 1. Longitudinal comparison of whole-body PET/CT findings, SUVmax, and Deauville scores across serial examinations.** | | | |
| --- | --- | --- | --- |
| **Examination Time** | **Lesion Location** | **SUVmax** | **Deauville Score** |
| Initial onset (2020-06-22) | Spleen | 6.5 | 4 |
| Second relapse  (2025-04-01) | Right lower leg skin | 4.7 | X |
| **Note:** SUVmax, maximum standardized uptake value. | | | |

| **Supplementary Table 2.Comparison of the patient’s clinical and radiological features across three episodes against the 2017 Tobin diagnostic criteria for CLIPPERS.** | | | | | |
| --- | --- | --- | --- | --- | --- |
| **Tobin 2017 CLIPPERS Criteria** | | **Initial Onset** | **First Relapse**  **(4 years later)** | | **Second Relapse**  **(10 months later; i.e., 5 years after the first episode)** |
| Clinical Presentation | | | | | |
| Subacute pontocerebellar dysfunction, with or without other CNS symptoms such as cognitive dysfunction and myelopathy. | | No (Fatigue, memory loss, hypersomnia, reduced speech, vomiting) | Yes | | Yes |
| CNS symptoms responsive to corticosteroid therapy. | | Yes | Yes | | Yes |
| Absence of peripheral nervous system disease. | | Yes | Yes | | Yes |
| Lack of alternative better explanation for clinical presentation. | | Yes | Yes | | Yes |
| MRI Features | | | | | |
| Homogenous, gadolinium enhancing nodules without ring enhancement or mass effect predominating in the pons and cerebellum, measuring ＜ 3 mm in diameter. | | No (Cerebellar and pontine involvement with supratentorial predominance.) | Yes | | Yes |
| Marked improvement in abnormal gadolinium enhancement with corticosteroid treatment. | | Yes | Yes | | Yes |
| Homogenous T2 signal abnormality where degree of T2 does not signiﬁcantly exceed the size of the area of post gadolinium enhancement. | | No (T2/FLAIR hyperintensity extended beyond the enhancing lesions) | Yes | | Yes |
| Spinal cord lesions with similar T2 and gadolinium enhancing lesions as above. | | Yes | No (No spinal cord lesions) | | No (No spinal cord lesions) |
| Neuropathology (Brain Biopsy) | | | | | |
| Dense lymphocytic inﬂammation with perivascular predominance and parenchymal diffuse inﬁltration; both white matter and grey matter could be involved. | | Not available | Not available | | Not available |
| T cells predominating inﬁltration (CD4^+^/ CD8^+^) with variable macrophage components. | | Not available | Not available | | Not available |
| Absence of myelin loss or focal secondary myelin loss. | | Not available | Not available | | Not available |
| Lack of alternative better explanation for pathological presentation. | | Not available | Not available | | Not available |
| ***Note:** Deﬁnite CLIPPERS, Patient fulﬁlling all clinical, radiological criteria, neuropathological criteria.  Probable CLIPPERS, Patients fulﬁlling all clinical and radiological criteria without available neuropathology  The spinal cord involvement row is not a mandatory criterion in the Tobin et al. (2017) diagnostic framework. Therefore, the "No" entry at relapse in this row should not be interpreted as a failure to meet the required criteria for probable CLIPPERS. | | | | | |
| Supplementary Table 3. Differential diagnosis of CLIPPERS | | | | | |
| **Differential diagnosis** | **Distinctive features** | | | **Reason for exclusion / low likelihood** | |
| MOGAD | Optic neuritis; ADEM-like lesions; longitudinal extensive transverse myelitis; MOG-IgG positive | | | Lack of typical clinical and MRI features; low-titer, transient MOG-IgG positivity; CSF MOG-IgG negative | |
| GFAP astrocytopathy | Radial perivascular enhancement on MRI; meningitis/encephalitis or myelitis; CSF lymphocytic pleocytosis with elevated protein, GFAP-IgG positivity in CSF | | | Absence of characteristic linear perivascular radial enhancement; GFAP-IgG negative in CSF and serum | |
| CTCL with secondary CNS involvement | Rare late CNS relapse; presents with altered mental status, motor dysfunction, gait disturbance, and cranial nerve palsies. Brain MRI may be normal or show T2 hyperintense lesions in the pons and right middle cerebellar peduncle. CSF PCR for T-cell receptor (TCR) gene rearrangement may detect clonal T cells and aid diagnosis. Poor prognosis (median survival ~5 months). Isolated CNS involvement may occur. | | | Serial PET/CT showed no systemic recurrence; skin and CSF TCR gene rearrangement analyses were negative; 5-year follow-up showed no progressive lymphoma; MRI evolved toward the characteristic CLIPPERS pattern | |
| Primary CNS lymphoma | DWI restriction (low ADC); ring/homogeneous enhancement with mass effect; MRS lipid/lactate peaks; hypermetabolic PET/CT lesions; biopsy-proven lymphoma and positive CSF TCR/B-cell clonality | | | No diffusion restriction or mass effect on MRI; repeated PET/CT negative; CSF TCR rearrangement negative | |
| CNS vasculitis | Stroke-like episodes; multifocal cerebral infarcts; vascular abnormalities on MRA | | | No multifocal infarcts; MRA showed no abnormalities | |
| Neurosarcoidosis | Leptomeningeal enhancement; cranial neuropathies; systemic sarcoidosis; elevated serum ACE; non-necrotizing granulomas | | | No systemic sarcoidosis; no leptomeningeal enhancement; no cranial neuropathies; no supportive laboratory or pathological evidence | |
| AE | Cognitive impairment; limbic encephalitis; neuropsychiatric symptoms, neuronal autoantibodies may be negative in seronegative cases | | | MRI showed predominant supratentorial white matter and brainstem involvement rather than limbic/mesial temporal lobe; neuronal autoantibody panel negative in serum and CSF; CSF OCB negative | |
| Intravascular lymphoma | Subacute encephalopathy or stroke-like episodes; multifocal white matter lesions; diffusion restriction on MRI; high FDG uptake on PET/CT; elevated LDH; possible skin or multi-organ involvement; diagnosis requires biopsy | | | No systemic or cutaneous involvement; no diffusion-restricted infarct-like lesions on DWI; no progressive or evolving lesions during 5-year follow-up | |

***Note:** CLIPPERS, chronic lymphocytic inflammation with pontine perivascular enhancement responsive to steroids; MOGAD, myelin oligodendrocyte glycoprotein antibody–associated disease; GFAP astrocytopathy, glial fibrillary acidic protein–associated autoimmune astrocytopathy; AE, autoimmune encephalitis;ADEM, acute disseminated encephalomyelitis; TCR, T-cell receptor; CNS, central nervous system; MRI, magnetic resonance imaging; MRA, magnetic resonance angiography; PET/CT, positron emission tomography/computed tomography; DWI, diffusion-weighted imaging; ADC, apparent diffusion coefficient; CSF, cerebrospinal fluid; OCB, oligoclonal bands; LDH, lactate dehydrogenase; MRS, magnetic resonance spectroscopy; ACE, angiotensin-converting enzyme; FDG, fluorodeoxyglucose.

| **Supplementary Table 4.Literature review of reported CLIPPERS cases with MOG-IgG positivity, lymphoma association, and other mimics** | | | | | | | | |
| --- | --- | --- | --- | --- | --- | --- | --- | --- |
| **Study** | **Patient (age/sex)** | **Etiology / Association** | **Clinical Presentation** | **the 2017 Tobin diagnostic criteria** | **Final Diagnosis** | **Treatment** | **Relapsed** | **Follow up (months)** |
| Obeidat et al., 2021 (1) | Case 1:Young male | MOG-lgG 1:10000(Serum, FACS) | Ataxia, vertigo, face numbness, paraparesis | Met (probable) | MOGAD | Steroids, IVIG  Rituximab+Mycophenolate | Yes | 48 |
|  | Case 2: Middle age female | MOG-lgG 1:1000(Serum, FACS) | Ataxia Vertigo paraparesis | Met (probable) | MOGAD | Steroids, IVIG Rituximab | Yes | 70 |
|  | Case 3: Young female | MOG-lgG 1:100(Serum, FACS) | Ataxia, diplopia, nausea, vomiting | Met (probable) | MOGAD | Steroids, Plasmapheresis,  Rituximab | Yes | 24 |
|  | Case 4: Young female | MOG-lgG 1:1000(Serum, FACS) | Weakness and visual loss (optic neuritis) | Met (probable) | MOGAD | Steroids, Mycophenolate | No | 12 |
| Symmonds et al., 2015 (2) | 36 / Female | MOG-lgG positive (Serum, CBA, titer not specified) | Initial (at onset): Dizziness, left facial paresthesia, dysarthria, gait ataxia, diplopia. Relapse (5 months later): Progressive leg tightness, altered perineal sensation, spastic paraparesis, urinary retention. | Met (probable) | MOGAD | Initial:High-dose IV Methylprednisolone, then oral Prednisolone (1 mg/kg/day) tapered over 5 months.Relapse: Further pulsed IV steroids and maintenance oral steroids. | Yes | 7 |
| Ferilli et al., 2022 (3) | 35 / Male | MOG-lgG 1:480(Serum, CBA) | Headache, unsteadiness and blurred vision(optic neuritis) | Met (probable) | MOGAD | Intravenous and oral steroids | Yes | 59 |
| Guo et al., 2022 (4) | 25 / Male | NMDAR‑Ab: Fluctuated in CSF (1:1 to 1:320) and serum (up to 1:32). MOG‑Ab: Serum positive only at the last episode (titer 1:10) | Initial: fever, headache, agitation, delirium, orofacial involuntary movements, seizures. Later: diplopia, ataxia, optic neuritis, limb weakness, brainstem symptoms (vertigo, dysarthria, facial palsy), and transverse myelitis. | Met (probable) | MNOS (Anti‑NMDAR encephalitis + MOG‑IgG associated disease) | High‑dose IV methylprednisolone, IVIG, oral prednisone, mycophenolate mofetil, rituximab (later discontinued). | Yes | 59 |
| Sagawa et al., 2026 (5) | Case 1: 23  / Female | Compound heterozygous UNC13D variants: c.2588G>A (p.G863D) and c.3229_3235del (p.R1077Sfs*48); FHL3 with mildly decreased NK degranulation and reduced Munc13‑4 expression | Initial (age 15): dysarthria, vertigo; spontaneous cerebellar lesions. Age 16: fever, cytopenia, hepatosplenomegaly, pulmonary infiltrates. Age 19–21: recurrent diplopia, headache, impaired consciousness, visual acuity loss; MRI showed punctate‑to‑nodular enhancing lesions in brainstem, cerebellum, and cerebrum, typical of CLIPPERS. | Met (probable) | FHL3 (familial hemophagocytic lymphohistiocytosis type 3) with CLIPPERS‑like syndrome | Corticosteroids (mPSL pulse, oral PSL); CHOP chemotherapy (for suspected lymphoma); subsequent allogeneic HCT from HLA‑matched sibling | Yes | 30 |
|  | Case 2: 18 / Female | Compound heterozygous UNC13D variants: c.2588G>A (p.G863D) and c.118‑307G>A (deep intronic); FHL3 with mildly decreased NK degranulation and reduced Munc13‑4 expression | Onset at 11 y: ataxia, dysarthria, intention tremor, nystagmus, diplopia. Later developed motor paralysis and sensory impairment of lower limbs due to spinal cord lesions. MRI showed multiple punctate/nodular enhancing lesions in cerebellum and brainstem. | Met (Definite, brain biopsy confirmed perivascular CD4+ T‑cell infiltration) | FHL3 with CLIPPERS-like syndrome | Corticosteroids, IVIg, cyclophosphamide, methotrexate, azathioprine, rituximab, tacrolimus; then allogeneic HCT from unrelated donor | No | 12 |
| Galazky et al., 2022 (6) | 51  / Male | CNS diffuse large B‑cell lymphoma (biopsy‑proven) | Progressive left‑sided ataxia, gait unsteadiness, dysarthria, diplopia, mild right facial paresis. Later (after 6 months) accelerated ataxia, dysphagia, tetraparesis, nursing‑care dependence. | Met (probable) | Primary CNS B‑cell lymphoma (high‑grade malignant B‑cell lymphoma; CD20+/CD79a+, high proliferation index) | Initial: IV methylprednisolone pulses (repeated) with good temporary response. Later: methotrexate as maintenance. After biopsy diagnosis: rituximab + methotrexate (hemato‑oncological treatment). | Not applicable | 10 |
| Shervedani et al., 2024 (7) | 47  / Male | Hodgkin's lymphoma (HL) – history of mediastinal HL treated with chemotherapy 11 years prior to CLIPPERS onset | 4‑month progressive dizziness, ataxia, diplopia, left‑sided numbness and paresthesia, dysarthria, end‑gaze nystagmus, impaired tandem gait. | Met (probable) | lymphoma-associated CLIPPERS | Initial: IV methylprednisolone 1 g/day × 4 days, then oral prednisolone 25 mg/day (tapered). Relapse: plasmapheresis (10 L total exchange) followed by oral prednisolone 25 mg/day. | Yes | 12 |
| Yang et al., 2024 (8) | 32 / Female | No | Subacute dizziness, dysarthria, unsteady gait, progressive headache, and memory loss. No facial paresthesia, diplopia, or urinary incontinence. | Met (Definite, biopsy‑proven perivascular lymphocytic infiltrates) | CLIPPERS | Intravenous methylprednisolone (1 g/day × 5 days), followed by prolonged oral prednisone (tapered to 30 mg/day) combined with mycophenolate mofetil (0.5 g twice daily) | No | 12 |
| Encarnacion et al., 2025 (9) | 54  / Male | No | 1‑month progressive diplopia, blurred vision, facial paresis, and gait instability (lower limb weakness). | Met (probable) | Probable CLIPPERS | Initial: IV methylprednisolone (1 g/day × 5 days) + IVIG (0.4 g/kg/day × 5 days). Then oral prednisolone (70 mg/day, gradually tapered over months). | No | 1 |
| Olejnik et al., 2025 (10) | 65  / Male | No | 5‑week progressive diplopia, dizziness, recurrent headaches, bilateral facial tingling. On admission: left lateral rectus paresis, convergent strabismus, horizontal nystagmus, positive Romberg test. Later worsened with ataxia of left lower limb. | Met (probable) | Probable CLIPPERS | IV methylprednisolone (total 7 g over 7 days), followed by oral prednisone (starting 64 mg/day, tapered gradually). | No | 3 |
| Zhang et al., 2021 (11) | 43  / Female | No | 3‑month progressive paresthesia in both lower limbs (subjective loss of limb awareness but normal sensation), double vision (worse on left gaze, exacerbated by fatigue), and gait unsteadiness (needed support to stand). | Met (probable) | Probable CLIPPERS | IV methylprednisolone 500 mg/day for 5 days, then gradually tapered to oral dose. | No | ＞12 |
| Meller et al., 2023 (12) | 18  / Female | No | Initial (2016): progressive lower limb weakness over 1 year, leading to spastic tetraparesis (lower limbs > upper), bilateral Babinski sign, no sensory loss or sphincter dysfunction. Relapse (2021): worsening gait disability, spastic tetraparesis, sphincter dysfunction; MRI showed innumerable punctate enhancing lesions in pons, medulla, and entire spinal cord (CLIPPERS pattern). | Met (probable) | Probable CLIPPERS | Initial (2017): IV methylprednisolone (5 boluses) followed by oral prednisone (50 mg/day tapering) + azathioprine (100 mg/day). Discontinued after ~3 years (2020). Relapse (2021): IV methylprednisolone (total 5 g) followed by oral prednisone (40 mg/day, tapered to 20 mg) + azathioprine (100 mg/day) maintained. | Yes | 60 |
| Lebel et al., 2020 (13) | 59 / Male | Primary cutaneous anaplastic large cell lymphoma (ALCL), CD30+, ALK– | Memory impairment, dizziness, brain MRI showed a large left frontal mass with significant edema, with vitreous involvement (TCR rearrangement positive) | N/A | CNS recurrence of cutaneous ALCL | MATRix regimen (methotrexate, cytarabine, thiotepa) → ICE consolidation (ifosfamide, carboplatin, etoposide) + intrathecal and intravitreal injections → autologous stem cell transplantation | No | 36 |
| Preston et al., 2023 (14) | Case 1: 44 / Female | Mycosis fungoides (stage 1A, later large cell transformation) | Lethargy, right temporal lobe lesion on MRI with restricted diffusion and enhancement; also had imaging features suggestive of multiple system atrophy | N/A | CNS involvement by CTCL | Systemic corticosteroids; whole‑brain radiation considered but not given due to functional decline | no remission | 0.6 |
|  | Case 2: 41 / Male | Mycosis fungoides (stage 1B, later large cell transformation, CD30+) | Unsteady gait, speech difficulties; MRI showed left cerebellar mass with mass effect and leptomeningeal spread | N/A | CNS involvement by CTCL | Brentuximab vedotin + CHP, then high‑dose methotrexate + MCHOP; palliative care later | Progressed | 4.5 |
|  | Case 3: 66 / Female | Mycosis fungoides (stage 2B, later large cell transformation) | Right monocular visual deficits (intraocular involvement), then left upper arm weakness and paresthesia; MRI showed right parietal mass | N/A | CNS involvement by CTCL | Ocular radiation; subtotal resection; high‑dose methotrexate + temozolomide | Systemic relapse (skin and nodes) after ~3 years; no CNS relapse at that time | 48 |
|  | Case 4: 65 / Male | Sézary syndrome (stage IVA2) | Severe right maxillary and bilateral periorbital involvement with edema and visual changes; later bradykinesia, psychomotor slowing, cognitive decline (suggestive of CNS invasion) | N/A | Presumed CNS involvement by CTCL (no confirmatory imaging before death) | Palliative‑intent radiation to periorbital area | Progressed | 6.2 |
| ***Note:**  ¹ Abbreviations: MOG‑IgG = myelin oligodendrocyte glycoprotein immunoglobulin G; FACS = fluorescence‑activated cell sorting; CBA = cell‑based assay; FHL3 = familial hemophagocytic lymphohistiocytosis type 3; HCT = hematopoietic cell transplantation; IVIG = intravenous immunoglobulin; MNOS = overlapping anti‑NMDAR encephalitis and MOGAD; NMDAR = N‑methyl‑D‑aspartate receptor; mPSL = methylprednisolone; PSL = prednisolone; CNS = central nervous system; HL = Hodgkin’s lymphoma; N/A = not applicable  ² Diagnostic criteria according to Tobin et al. (2017): “Met (probable)” indicates that clinical and radiological features are met without histopathological confirmation (no brain biopsy performed or biopsy non‑diagnostic); “Met (Definite)” indicates biopsy‑proven perivascular lymphocytic infiltration consistent with CLIPPERS pathology.  ³ Follow‑up duration: “＞12” indicates more than 12 months, with the exact duration not further specified in the original report.  ⁴ Relapse: “Yes” indicates at least one clinical or radiological relapse during the follow‑up period; “No” indicates no relapse; “Not applicable” refers to cases where the final diagnosis was a malignancy (e.g., CNS lymphoma) rather than CLIPPERS, so the concept of CLIPPERS relapse does not apply.  ⁵ Etiology / Association: For MOG‑IgG‑positive cases, the antibody titers and detection methods are provided when available in the original reports. For lymphoma‑associated cases, the type of lymphoma and its temporal relationship to CLIPPERS onset are noted. | | | | | | | | |

**Reference**

1	Obeidat AZ, Block AN, Hooshmand SI. "Peppering the pons": CLIPPERS or myelin oligodendrocyte glycoprotein associated disease? Mult Scler Relat Disord. 2021;51:102874.

2	Symmonds M, Waters PJ, Küker W, Leite MI, Schulz UG. Anti-MOG antibodies with longitudinally extensive transverse myelitis preceded by CLIPPERS. Neurology. 2015;84(11):1177–9.

3	Ferilli MAN, Papi C, Sabatelli M, Colosimo C, Iorio R. MOG autoimmunity mimicking CLIPPERS syndrome: Case report and literature review. J Neuroimmunol. 2022;367:577875.

4	Guo J, Bu Y, Liu W. Case Report: A Case With MOGAD and Anti-NMDAR Encephalitis Overlapping Syndrome Mimicing Radiological Characteristics of CLIPPERS. Front Immunol. 2022;13:832084.

5	Sagawa H, Hirata K, Katayama S, Shibata H, Toyofuku E, Kaneko S, et al. Two Cases of CLIPPERS-like Syndrome Sharing a Hypomorphic UNC13D Variant. J Clin Immunol. 2026;46(1).

6	Galazky I, Büntjen L, Voges J, Sandalcioglu IE, Mawrin C, Haghikia A. Brain biopsy in patients with CLIPPERS syndrome: why and when. Ther Adv Neurol Disord. 2022;15:17562864211062821.

7	Shervedani AK, Tabibian F, Khotbesara MG, Adibi I. Chronic lymphocytic inflammation with pontine perivascular enhancement responsive to steroids triggered by Hodgkin's lymphoma: A case report and brief literature review. Clin Case Rep. 2024;12(1):e8377.

8	Yang Y, Li S, Yang Y, Zeng T, Li J, Tang Y. Immunosuppressive Therapy Reversing Obstructive Hydrocephalus in CLIPPERS. Neurology. 2024;102(9):e209396.

9	Encarnación JA, Hernández AL, López GL, Alonso-Romero JL, De la Fuente Muñoz MI, Cánovas EC, et al. Probable chronic lymphocytic inflammation with pontine perivascular enhancement responsive to steroids syndrome: management with corticosteroids and intravenous immunoglobulin-a case report. J Med Case Rep. 2025;19(1):351.

10	Olejnik P, Szyluk B, Zakrzewska-Pniewska B, Kostera-Pruszczyk A, Golenia A. CLIPPERS syndrome: a case report and differential diagnosis review. Postep Psychiatr Neurol. 2025;34(2):130–7.

11	Zhang L, Zhao D, Li J, Feng J, Zhang Q, Liu L, et al. A case report of CLIPPERS syndrome and literature review. Medicine (Baltimore). 2021;100(22):e26090.

12	Meller A, Pawlukowska W, Machowska-Sempruch K, Marta M. Spectrum of Autoimmune Diseases-Chronic Lymphocytic Inflammation with Pontine Perivascular Enhancement Responsive to Steroids (CLIPPERS)-Clinical Case. Medicina (Kaunas). 2023;59(3).

13	Lebel E, Benshushan S, Paltiel O. From the Skin Straight to the Brain: Rare CNS Recurrence of Cutaneous Anaplastic Large Cell Lymphoma. JCO Oncol Pract. 2020;16(5):247–8.

14	Preston JD, Jansen CS, Kosaraju S, Niyogusaba T, Zhuang TZ, Iwamoto SW, et al. Cutaneous T-cell lymphoma with CNS involvement: a case series and review of the literature. CNS Oncol. 2023;12(4):Cns105.
